# Draft Genome Sequences of Enterococcus faecium, Enterococcus gallinarum, and Lactococcus lactis Strains Isolated from a Mastitis-Infected Camel in Isiolo County, Kenya

**DOI:** 10.1128/mra.01083-22

**Published:** 2022-12-15

**Authors:** Monicah Maichomo, Rachael Gachogo, Ednah Masila, Irene Ogali, Leon Otieno, Nathan Langat, Ruth Onywera, Vincent Malonza, Christine Inguyesi, Frank Onyambu, Nimmo Gicheru, Hezron Wesonga, Edwin Murungi

**Affiliations:** a Veterinary Research Institute, Kenya Agricultural and Livestock Research Organisation, Nairobi, Kenya; b Division of Immunology, Department of Human Pathology, University of Cape Town, Cape Town, South Africa; c Centre for Molecular Biosciences and Genomics, Nairobi, Kenya; d Department of Clinical Medicine and Therapeutics, University of Nairobi, Nairobi, Kenya; e School of Health Sciences, Meru University of Science and Technology, Meru, Kenya; f International Livestock Research Institute, Nairobi, Kenya; g Department of Medical Biochemistry, School of Health Sciences, Kisii University, Kisii, Kenya; University of Maryland School of Medicine

## Abstract

We report the draft genome sequences and annotation of Enterococcus faecium, Enterococcus gallinarum, and Lactococcus lactis isolates that were recovered from a mastitis-infected camel in Isiolo County, Kenya. Collectively, these data provide an invaluable repository for data mining to support the development of a potential multicomponent mastitis subunit vaccine.

## ANNOUNCEMENT

Globally, the economic losses arising from mastitis are colossal and continue to rise due to the increase in antimicrobial resistance (1). Camels (Camelus dromedaries) are vital dairy animals in the vast arid and semiarid regions of northern Kenya, and camel mastitis seriously endangers the already drought-ravaged livelihoods of pastoralists in these regions. The *Enterococcus* and *Lactococcus* genera have been implicated in the causation of mastitis (2, 3); therefore, development of an efficacious multicomponent mastitis subunit vaccine is an urgent priority.

We report the draft genome sequences of Enterococcus faecium, Enterococcus gallinarum, and Lactococcus lactis isolates that were recovered from a mastitis-infected camel in Isiolo County, Kenya. Milk samples were inoculated on blood agar containing 5% sheep blood and were incubated overnight at 37°C. Colonies with zones of hemolysis that were catalase negative and Gram positive were cultured in Edward’s selective medium to ascertain genus type (4). Lancefield grouping confirmed the isolates as group D (enterococci) (5). M-17 agar medium was used to confirm L. lactis colonies (6). The isolates were cultured on nutrient agar plates by incubation under aerobic conditions at 37°C for 16 h, followed by enrichment of fresh single colonies in 100 mL nutrient broth with aerobic incubation at 37°C for 16 h, with rotation at 200 rpm (7), to an optical density at 600 nm (OD_600_) of 0.5 to 0.7 (8). The cultures were used for DNA extraction.

Genomic DNA was extracted using the QIAamp DNA kit (Qiagen) according to the manufacturer’s instructions and was neither sheared nor size selected prior to Oxford Nanopore Technologies (ONT) library preparation using the manufacturer’s native barcoding expansion 1–12 kit (EXP-NBD104) in conjunction with the ligation sequencing kit (SQK-LSK109). Sequencing was performed on a MinION system using a R9.4.1 flow cell, and base calling and demultiplexing were undertaken using Guppy v3.6.1 (https://mirror.oxfordnanoportal.com/software/analysis/ont-guppy-cpu_3.0.3_linux64.tar.gz). Adaptors were trimmed using Porechop v0.2.4 (9), and Nanofilt v2.5.0 (10) was used for quality filtering by removing reads of <500 bp and reads with average quality scores of <10. The draft L. lactis genome was assembled using Unicycler v0.4.9 (11), while the E. gallinarum and E. faecium genomes were assembled using Flye v2.9.1 (12). The quality of the assembled genomes was assessed using QUAST v5.2.0 (13). The contigs were annotated using NCBI PGAP v6.1 (14). The taxonomy of the organisms was ascertained using BLAST2seq v2.5 (15). Default parameters were used for all software except where otherwise noted. Assembly and annotation metrics are shown in Table 1. Notably, given that these are ONT-only assemblies, the assemblies contain a large number of frameshifted genes, as depicted in Table 1.

**TABLE 1:** Assembly and genome annotation metrics for E. faecium, E. gallinarum, and L. lactis isolates

Feature	Data for:		
	Enterococcus faecium	Enterococcus gallinarum	Lactococcus lactis
No. of reads	8,595	11,050	2,770
Read *N*_50_ (kb)	26	35	26
Genome size (Mbp)	2.64	3.43	2.74
GC content (%)	38.28	40.83	35.19
Sequencing coverage (×)	22	24	36
No. of contigs	2	11	6
*N*_50_ (bp)	2,455,210	3,141,075	2,560,212
Total no. of genes	2,631	3,935	3,191
Total no. of coding sequences	2,542	3,852	3,104
No. of protein-coding genes	1,329	2,432	2,276
No. of rRNAs	18	15	19
No. of tRNAs	67	64	64
No. of noncoding RNAs	4	4	4
No. of CRISPR sequences	0	0	0
No. of frameshifted pseudogenes/total no. of pseudogenes	1,174/1,213	1,335/1,420	777/828

This study was reviewed by the Kenya Agricultural and Livestock Research Organisation (KALRO) Institutional Animal Care and Use Committee (IACUC) (approval number KALRO/VSRI/IACUC2/12012017).

### Data availability.

This whole-genome shotgun project has been deposited in DDBJ/ENA/GenBank under accession numbers JAOPKV000000000, JAOTOH000000000, and JAOTOI010000000. Raw sequence reads are available in the Sequence Read Archive (SRA) database under accession numbers SRR21729046, SRR21743019, and SRR21742702.
